# Impact of Overuse and Sexually Transmitted Infections on Seminal Parameters of Extensively Managed Bulls

**DOI:** 10.3390/ani11030827

**Published:** 2021-03-15

**Authors:** Giovanni Montoya-Monsalve, María-Jesús Sánchez-Calabuig, Javier Blanco-Murcia, Laura Elvira, Alfonso Gutiérrez-Adán, Priscila Ramos-Ibeas

**Affiliations:** 1 MSD Animal Heath, C/Josefa Valcárcel, 38, 28027 Madrid, Spain; giovanni.montoya.monsalve@merck.com (G.M.-M.); laura.elvira@merck.com (L.E.); 2 Departamento de Medicina y Cirugía Animal, Facultad de Veterinaria, UCM, Avda. Puerta de Hierro s/n, 28040 Madrid, Spain; msanch26@ucm.es; 3 Department of Animal Reproduction, National Institute for Agriculture and Food Research and Technology (INIA), Avda. Puerta de Hierro 12, Local 10, 28040 Madrid, Spain; agutierr@inia.es (A.G.-A.); priscilaramosibeas@gmail.com (P.R.-I.)

**Keywords:** extensively managed bull, seminal parameters, herd fertility, infectious bovine rhinotracheitis, bovine viral diarrhea, bull: cow ratio

## Abstract

**Simple Summary:**

Natural service is currently the predominant method of breeding in extensively managed beef herds. In these herds, the importance of the bull has been largely overlooked, focusing instead on female fertility. However, the bull might be the most important factor in determining the reproductive performance of the herd, because one subfertile or infertile bull could mean a decrease of 25 to 40 calves per year. Thus, bull management is critical to increase herd fertility. Infectious bovine rhinotracheitis (IBR) and bovine viral diarrhea (BVD) are infections associated with reduced conception rates. In this study, we analyzed the effect of IBR, BVD, and bull: cow ratio on seminal parameters of the bull and herd fertility, finding a significant negative effect of BVD on sperm concentration and motility, and a negative correlation between the number of cows per bull and herd fertility. Our data suggest that serological control of BVD and sperm parameters, as well as including less than 40 cows per bull, could improve the reproductive efficiency of the herd in extensively managed herds.

**Abstract:**

Natural service remains the main breeding method in extensively managed beef herds. Although the bull might be the most important factor in determining herd fertility, its importance has been largely overlooked, focusing instead on female fertility. Management of the bull is critical to maximize the opportunities for cow conception. Infectious bovine rhinotracheitis (IBR) and bovine viral diarrhea (BVD) are infections associated with reduced conception rates. This study aimed to determine the effect of both IBR and BVD infection, and bull: cow ratio on seminal parameters in the bull and herd fertility. The presence of antibodies to IBR and BVD, seminal parameters (volume, concentration, mass, and progressive motility), and herd fertility were analyzed in 158 extensively managed bulls. Sperm concentration and mass motility, as well as herd fertility, were significantly lower in BVD-positive bulls. No significant differences were found between IBR-positive and -negative bulls in any reproductive parameter. Sperm concentration was negatively affected by BVD infection in both Charolais and Limousin bulls, whereas mass motility and herd fertility were reduced in Limousin bulls only. No differences were observed in the cow: bull ratio between BVD+ and BVD- bulls. A significant negative correlation was detected between the number of cows per bull and herd fertility, which was negatively affected when herds had more than 40 cows per bull. In conclusion, BVD and bull overuse negatively affect the reproductive performance of the herd.

## 1. Introduction

Extensive cattle production generates enormous wealth, yielding economic benefits, employment, and environmental sustainability [1]. Fertility in extensive cattle production is determined by different factors such as the nutrition and body state of both males and females. Poor nutrition or body condition in ungulates can adversely affect hypothalamic-pituitary function [2], delay puberty, prevent ovulation [3], and reduce pregnancy rates and offspring production [4]. Moreover, it is mandatory that bulls maintain a good nutrition plan during the rest period and are supplemented, if necessary, to ensure an adequate body condition for the following breeding season [5]. Nowadays, fertility assessments are failing in extensive beef cattle systems, leading to a decrease of up to 80% in the number of bulls allowed to serve cows [6,7]. Moreover, in Europe, the average annual herd fertility, understood as the percentage of cows calved during a year, is near 85%, whereas in Spain it is around 70% [8]. This low extensive herd fertility is most likely due to the fact that the importance of the bull has been largely overlooked, with attention being predominantly focused on female fertility. However, the bull might be the most important factor in determining the reproductive performance of the herd, because one subfertile or infertile bull could mean a significant decrease in calves per year [5].

In contrast to intensively managed cattle, in which artificial insemination was introduced in the 1950s, displacing natural service as the selected breeding method, logistical difficulties have limited its use in extensively managed herds [9]. Thus, natural service has remained the predominant technique for breeding in such herds, making it mandatory that bulls, which represent the most important factor when no artificial insemination is performed, have good semen quality together with a good serving capacity and physical soundness [7]. 

Moreover, infectious diseases, such as bovine viral diarrhea (BVD), infectious bovine rhinotracheitis (IBR), and infection by Besnoitia, Trichomonas, Neospora, and Campylobacter, have also been linked to fertility and reproductive disorders in this species, causing an important economic impact in the livestock industry [10,11]. Particularly, BVD and IBR, caused by BVD virus (BVDv) and bovine herpesvirus 1 (BoHV-1), respectively, are highly contagious diseases of high economic and trade importance [12]. Both cause suboptimal reproductive efficiency in herds; thus, serological testing is crucial to examine the extent and importance of these diseases [13,14]. Acute BVD infections are usually mild or unapparent; however, reproductive problems associated with this disease are frequently missed when other clinical signs are not present [15]. Furthermore, the trans-placental transmission of BVDv can lead to the birth of persistently infected (PI) animals, which significantly contribute to the continuous spread of the virus through the herd [16]. Despite the fact that viral transmission from prolonged BVDv infected bulls appears to be low [17,18] and PI bulls can produce semen of appropriate quality [19,20], they are often associated with poor fertility [15]. 

On the other hand, IBR is another widely recognized cause of abortion, infertility, and suboptimal productivity in the herd [21,22]. Infected animals remain latently infected during their whole life. Subsequently, the virus can be reactivated by different causes of stress such as delivery or transport, and these animals eliminate the virus into the environment, which may lead to the infection of new animals and a new outbreak. For this reason, latently infected animals are the main form of maintenance of infection within the herd [10]. Nowadays in Europe, there are officially IBR-free countries such as Switzerland, Austria, Norway, Sweden, and Finland; while others have National or Regional Eradication Plans such as France, Germany, Ireland, the Netherlands, the Czech Republic, and Hungary. Moreover, the European Union (EU) has legislation regarding IBR through Decision 2007/584/EC by which Directive 64/432/EEC is applied. In Spain, a new IBR prevention, control, and eradication plan has been recently implemented through RD 554/2019.

The number of cows per bull represents another important parameter to evaluate in extensively managed herds in order to obtain better reproductive performance. The percentage of bulls per cow is very variable between herds. Some studies have estimated that it ranges from 1.5% to 10% of bulls in different herds and that 2% might be enough to obtain successful pregnancy rates [23,24]. These data are consistent with other reports, which also highlight the importance of including semen analysis in the assessment of the players, classifying the bulls into fertile, subfertile, infertile, and sterile [25]. Thus, the present study aimed to investigate the seroprevalence status of IBR and BVD, and to analyze the sperm parameters in a large cohort of extensively managed bulls in Spain, as well as to determine the effects of both IBR and BVD infections, and the bull: cow ratio on seminal parameters and herd fertility in extensively managed bulls.

## 2. Materials and Methods 

### 2.1. Selection of Animals

The study was conducted between May and August 2014 and 2015 and involved 158 bulls (66 Charolais, 65 Limousin, and 27 of different autochthonous breeds) and 4299 cows that were randomly selected in two adjacent areas in Spain (Cáceres (Extremadura) and Salamanca (Castilla y León)). No fertility evaluations had been performed on the bulls prior to the start of the study. All procedures were approved by the Spanish Ethics Committee of Animal Welfare (RD 53/2013). Twenty-one farms that did not vaccinate the animals for IBR and BVD were included in the study to avoid interferences in the analyses. The bulls included in the study tested negative for Trichomonas and Campylobacter and their age ranged from 1 to 10 years old (mean of 4.06 years old). The average size of the cow herd was 30.49 ± 0.68 (mean ± SEM), ranging from 15 to 51 cows per bull. No differences were observed in bull: cow ratio between different farms (One-way ANOVA).

### 2.2. Herd Management and Fertility Data

Animals were fed on natural pastures supplemented with water, feed additives, and good quality straw in the summer season. Only cows in good physical condition were considered to analyze cow: bull ratio and herd fertility. Fertility was evaluated based on calving rate, which was assessed as the percentage of calving cows within the herd per year. Data were collected by both veterinarians and farm owners.

### 2.3. Sample Collection

Bulls included in the study were kept in sexual rest for 2 weeks. They were then held for semen collection in a handling sleeve. A transrectal massage was performed with a flattened hand. Feces were manually removed and electroejaculation was performed using a 75 mm diameter rectal probe with three ventrally oriented longitudinal electrodes (Pulsator IV; Lane Manufacturing, Denver, CO, USA). During semen collection, the pre-seminal fraction was discarded. The electroejaculator was set on automatic mode; one circuit contained a series of 40 cycles, with each cycle delivering a slightly higher intensity voltage. Each cycle lasted for 2 s followed by 2 s pause. Once a minimum sample was obtained (3 mL), the stimulation stopped. This procedure was carried out by a veterinarian, following the same protocol in all bulls included in the experiment.

### 2.4. Semen Analysis

Immediately after collection, semen samples were analyzed. Each sample was processed for volume, concentration, mass motility, and progressive motility. The volume of the sample was determined using a graduate micropipette (Gilson, Gilson International, Villiers-le-Bel, France). The sperm concentration was determined using a photometer (Accured Photometer, IMV, Huelva, Spain). Mass and progressive motility were subjectively evaluated by the same researcher for all analyses included in this study. Mass and progressive motile sperm were subjectively determined under phase-contrast microscopy at 400× magnification (B-500 TpH OPTIKA), using a scale ranging from 1 to 4, in which 1 = 0–39%, 2 = 40–59%, 3 = 60–79%, and 4 = ≥80% spermatozoa showed total or progressive motility. 

### 2.5. IBR and BVD Assays

Peripheral blood samples were obtained from the bulls included in this study. Samples were then allowed to clot, and the serum was separated by centrifugation at 1500 *g* for 10 min, and frozen at −20 °C until use. Serum samples were analyzed for antibodies against IBR virus and BVDv p80 (nonstructural protein 3; NS3NS3) by ELISA as previously described with some modifications [13]. 

### 2.6. Statistical Analysis

The differences in seminal parameters (concentration, volume, mass, and progressive motility) and herd fertility between infected and non-infected animals were analyzed by *t*-test. The differences in BVD and IBR prevalence between breeds were analyzed by Chi-square test. The differences in seminal parameters (concentration, volume, mass, and progressive motility) and herd fertility between breeds were analyzed by One-way ANOVA. To exclude bull age as a possible variant affecting the results, *t*-test was performed between infected and non-infected animals. To exclude a farm effect on bull: cow ratio, One-way ANOVA was performed. Linear regression and correlation analyses were performed between cow: bull ratio and reproductive parameters. All statistical analyses were performed with Prism GraphPad software (San Diego, CA, USA) and a value of *p* < 0.05 was considered significant.

## 3. Results

Bull age was excluded as a variable that could affect the results because no statistically significant differences were found between BVD+ (3.35 ± 0.32 years) and BVD- bulls (4.06 ± 0.18 years); or between IBR+ (3.67 ± 0.26 years) and IBR- bulls (3.99 ± 0.19 years) (*t*-test).

### 3.1. Correlation Between Bovine Viral Diarrhea (BVD) and Bull Fertility

**Figure 1:**
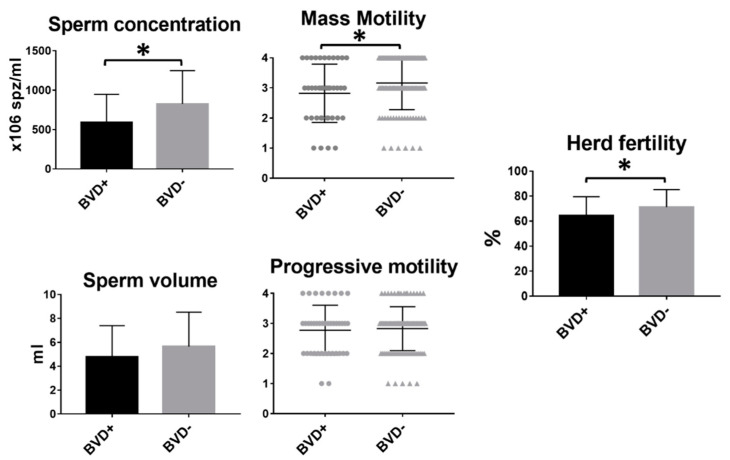
Differences in sperm parameters and fertility between bovine viral diarrhea (BVD)-positive and BVD-negative bulls. Mean and SEM of seminal parameters and herd fertility of BVD-positive and -negative bulls. * indicates statistically significant differences, *t*-test (*p* < 0.05).

Forty-two out of 157 bulls (26.58%) tested positive for BVD. Bulls infected with BVD showed a significantly reduced sperm concentration (*p* = 0.002) and mass motility (*p* = 0.044) compared to their non-infected counterparts. Although volume and progressive motility were slightly lower in bulls infected with BDV, no significant differences were found between BVD-positive and -negative animals. However, fertility was significantly lower in those herds in which the bull was infected (65.43 ± 2.51 vs. 71.15 ± 1.39%, *p* = 0.01) (Figure 1). No differences were observed in the cow: bull ratio between BVD+ (30.34 ± 1.54) and BVD- (30.53 ± 0.77) bulls (*t*-test), which excludes a possible interaction of cow: bull ratio in these results. 

Considering the different breeds, no differences were found in BDV prevalence between Charolais (19/66; 28.79%), Limousin (17/65; 26.15%), and bulls from autochthonous breeds (6/27; 22.22%). When we analyzed whether BVD affected reproductive parameters differently in the main breeds, we found that while sperm concentration was negatively affected by BVD infection in Charolais bulls (*p* = 0.004), mass motility (*p* = 0.02) and herd fertility (*p* = 0.047) were significantly reduced only in Limousin bulls. No differences were found between BVD-positive and -negative bulls from autochthonous breeds for any of the parameters analyzed, although this could be due to the reduced number of animals in this group (Table 1).

### 3.2. Correlation Analysis between Infectious Bovine Rhinotracheitis (IBR) and Bull Fertility

**Figure 2:**
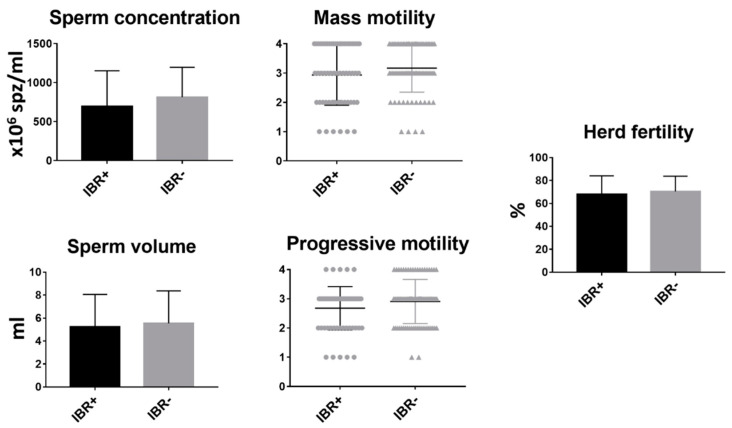
Analysis of sperm parameters and fertility between infectious bovine rhinotracheitis (IBR)-positive and IBR-negative bulls. Mean and SEM of seminal parameters and herd fertility of IBR-positive and IBR-negative bulls. * indicates statistically significant differences, *t*-test (*p* < 0.05).

Of the 158 bulls tested, 67 (42.40%) were positive for IBR. In this study, no correlation was found between the presence of IBR infection in the bull and the reproductive parameters evaluated (Figure 2).

When the effect of IBR in the different breeds was analyzed, no differences were found in the prevalence of IBR between Charolais (28/66; 42.42%), Limousin (29/65; 44.61%), and bulls from autochthonous breeds (9/27; 33.33%). Similarly, no differences were found in reproductive parameters between IBR-infected and non-infected bulls in the main breeds (Table 2). When we analyzed the differences in sperm parameters between breeds, sperm volume in Limousin bulls was significantly lower than in Charolais bulls (*p* = 0.001), and mass motility was significantly lower in Charolais bulls (*p* = 0.047) (Table 1 and Table 2).

### 3.3. Effects of Both IBR and BVD on Bull Fertility

**Figure 3:**
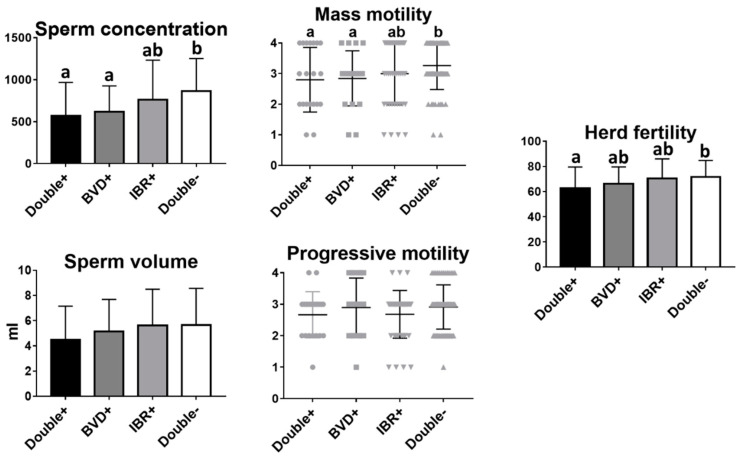
Analysis of sperm parameters and fertility in BVD- and/or IBR-positive and -negative bulls. Mean and SEM of seminal parameters and herd fertility of bulls infected with both BVD and IBR, BVD-only, IBR-only, and negative bulls. Different letters indicate statistically significant differences, One-way ANOVA (*p* < 0.05).

Next, we sought to find out whether infection with both BVD and IBR had an increased effect on reproductive parameters. Of the 157 bulls analyzed, 23 (14.56%) were positive for both IBR and BVD, 19 (12.02%) were only positive for BVD, 44 (27.85%) were only positive for IBR, and 72 (45.57%) were negative for both. Higher sperm concentration, mass motility, and herd fertility were found in bulls free of both IBR and BVD virus compared to animals positive for both diseases (*p* = 0.0102, *p* = 0.034 and *p* = 0.046, respectively). However, there were no statistically significant differences between animals infected with both BVD and IBR, and animals infected with only BVD or IBR (Figure 3).

### 3.4. Correlation between Cow: Bull Ratio and the Fertility of the Herd 

**Figure 4:**
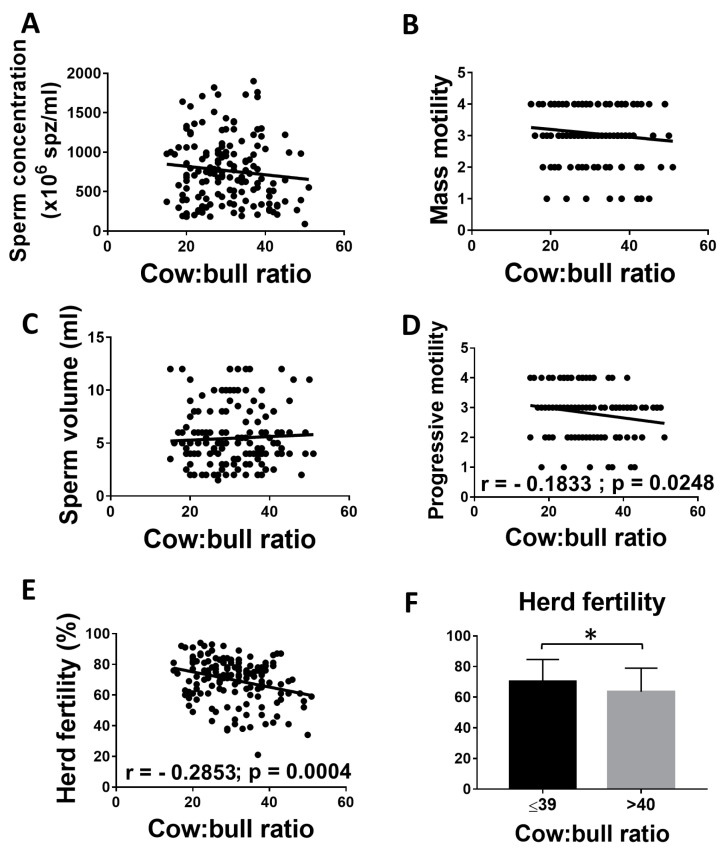
Correlation between cow: bull ratio and the fertility of the herd. (**A**–**E**) Linear regression between cow: bull ratio and reproductive parameters. (**F**) Mean and SEM of herd fertility of bulls with 40 or more cows and with less than 40 cows. * indicates statistically significant differences, *t*-test (*p* < 0.05).

A significant negative correlation (r = −0.1833) between progressive motility and the number of cows per bull (ranging from 15 to 51) was found. Although sperm concentration and mass motility were lower when the number of cows per bull increased, no differences were found between sperm volume, concentration, and mass motility and the cow: bull ratio (*p* > 0.05). However, there was a statistically significant negative correlation between herd fertility (r = −0.2853) and the number of cows per bull. Interestingly, when herds had more than 40 cows per bull, the negative effect on fertility was statistically significant (Figure 4F). A significant negative correlation between herd fertility and the number of cows per bull was also observed when only BVD-negative bulls were included in the analysis (r = −0.2936; *p* = 0.002), excluding a possible effect of BVD infection on this analysis.

## 4. Discussion

The present study reveals the high prevalence of IBR (42.40%) and BVD (26.58%) in extensively managed bulls in Spain and points out the negative effect of BVD on the reproductive parameters of the bull. Furthermore, we report that bull overuse decreases the reproductive efficiency of extensively managed herds, which is significantly reduced when more than 40 cows are allocated per bull. 

BVDv transmission within a herd or between herds depends primarily on contact between susceptible individuals and PI animals [26]. These individuals were likened to ‘Trojan Horses’ as they are virus-negative and antibody-positive, but they deliver PI calves [15]. Both acute and persistent infections can affect the reproductive soundness of bulls by reducing semen quality [26]. It has previously been shown that the virus causes prolonged testicular infection despite the presence of antibodies [27], and this is a potential concern in the herds that regularly use natural mating. In the present study, 26.58% of bulls tested positive for BVDv, which is a high incidence compared to other studies (0.4% in Irish dairy herds [12]). In our study, bulls infected with BVDv showed a significantly reduced sperm concentration and mass motility compared to their non-infected counterparts. In agreement with our results, it was previously reported that the reproductive performance [28] and semen quality from PI bulls may range from acceptable to abnormal, with various defects predominantly involving the head of the spermatozoa and low motility [29]. Similarly, it has been proven that there is a lower sperm concentration in bulls infected with BVDv [30]. This observation is in accordance with previous work highlighting that despite high viral titers, PI bulls can produce acceptable semen quality but are often associated with poor fertility [19,20]. Thus, it has been suggested that the removal of PI animals is the cornerstone for BVDv control and eradication, but enhancing herd immunity is also important [31,32]. Interestingly, it seems that this disease may have different effects depending on the breed. Although our study showed no differences in BDV prevalence between Charolais, Limousin, and bulls from autochthonous breeds, BVD infection negatively affected sperm concentration in both Charolais and Limousin bulls, but mass motility and herd fertility were reduced in Limousin bulls only. Further studies with a higher number of animals are required to confirm these results.

IBR infection is another widely recognized cause of abortion, infertility, and suboptimal productivity in the herd [21,22]. Moreover, BoHV-1 seroprevalence has been associated with herd size, though this may be a cluster risk factor [33]. In our study, BoHV-1 seroprevalence was 42.40%, much higher than other studies (16.7% in Irish dairy herds [12]). However, our results showed no differences in the sperm parameters evaluated between BoHV-1-positive and BoHV-1-negative bulls. A negative impact associated with IBR, regarding milk production [22] and respiratory diseases in the calves [34], has been previously reported. As a result, most of the countries in Europe have decided to establish programs for the prevention, control, and eradication of IBR (e.g., in Spain, through Royal Decree (RD) 554/2019). These programs have already shown interesting results in Germany, Hungary, and Italy [35]. Similar results were found by Raaperi et al. [34]. In this study, the IBR vaccination resulted in a reduction in the calving interval and showed a trend towards higher milk production and lower mortality from pneumonia in the calving. When we analyzed the effect of double infection with BVD and IBR on reproductive parameters, animals infected with both diseases showed lower sperm concentration, mass motility, and herd fertility than non-infected animals, but no significant differences were found between animals infected with both diseases and those infected only with BVD, suggesting that the negative effect on reproductive parameters could be explained by BVD infection only.

Interestingly, in our study, a high number of cows per bull appeared to directly affect reproductive parameters. A negative correlation (r = −0.2853) was found between the number of cows per bull and fertility. Moreover, fertility was significantly affected when herds were held with more than 40 cows per bull. Other studies have reported that pregnancy rates can be successful with ratios around 50 cows per bull; however, the authors point out that the bulls need to be of high reproductive performance [23,24]. Moreover, in a different study, the authors point out that introducing higher bull: cow ratios (ranging from 1:8 to 1:68) did not affect the return to oestrus pregnancy rate. Similarly, they found no correlation between the number of open cows per bull and return to oestrus conception rates [36].

## 5. Conclusions

This study provides an extensive analysis of the prevalence of BVD and IBR and of seminal parameters of extensively managed bulls in Spain. In the present study, no correlation was found between the presence of IBR infection in the bull and the reproductive parameters evaluated. Sperm concentration, mass motility, and herd fertility were negatively affected by BVD infection and by the overuse of bulls, decreasing the fertility of the herd. In order to improve the reproductive parameters of the herd, it seems mandatory to perform serological analyses for BVD and sperm analyses, as well as restricting the number of cows per bull to less than 40. However, further studies focusing on the number of cows per bull are necessary to narrow down the adequate ratio to improve the reproductive parameters of the herd.

## Figures and Tables

**Table 1:** Differences in sperm parameters and fertility between BVD-positive and -negative Charolais, Limousin, and autochthonous bulls.

Breed	n		Volume (ml)		Concentration (10^6^ spz/mL)		Mass Motility		Progressive Motility		Fertility (%)	
	BVD+	BVD-	BVD+	BVD-	BVD+	BVD-	BVD+	BVD-	BVD+	BVD-	BVD+	BVD-
Charolais	19	47	5.26 ± 0.68	6.71 ± 0.42 ^a^	564.8 ± 77.28	877.7 ± 59.03	2.39 ± 0.23 ^a^	2.82 ± 0.14 ^a^	2.58 ± 0.16	2.73 ± 0.12	64.74 ± 3.25	71.61 ± 2.02
Limousin	17	48	4.32 ± 0.57	4.34 ± 0.32 ^b^	640.2 ± 106.4	802.3 ± 65.61	3.2 ± 0.22 ^b^	3.47 ± 0.11 ^b^	2.93 ± 0.25	2.84 ± 0.08	63.06 ± 4.19	70.7 ± 1.92
Autochthonous	6	21	4.58 ± 0.81	6.45 ± 0.73 ^a^	547 ± 102	783.1 ± 92.71	3.17 ± 0.3 ^ab^	3.21 ± 0.2 ^ab^	3 ± 0.36	3.05 ± 0.19	67.33 ± 6.41	70.85 ± 3.82

Underlined values indicate statistically significant differences between infected and non-infected animals within each breed (*p* < 0.05, *t*-test). Different letters indicate statistically significant differences between breeds (*p* < 0.05, One Way ANOVA).

**Table 2:** Differences in sperm parameters and fertility between IBR-positive and IBR-negative Charolais, Limousin, and autochthonous bulls.

Breed	n		Volume (mL)		Concentration (10^6^ spz/mL)		Mass Motility		Progressive Motility		Fertility (%)	
	IBR+	IBR-	IBR+	IBR-	IBR+	IBR-	IBR+	IBR-	IBR+	IBR-	IBR+	IBR-
Charolais	28	38	6.30 ± 0.55 ^a^	6.27 ± 0.50 ^a^	751.1 ± 84.34	816.1 ± 63.22	2.50 ± 0.21 ^a^	2.83 ± 0.15 ^a^	2.60 ± 0.13	2.76 ± 0.14	69.37 ± 2.94	67.76 ± 2.17
Limousin	29	36	4.05 ± 0.42 ^b^	4.57 ± 0.37 ^b^	686.7 ± 91.73	820.6 ± 69.18	3.23 ± 0.18 ^b^	3.53 ± 0.11 ^b^	2.73 ± 0.14	2.97 ± 0.11	66.89 ± 2.90	70.26 ± 2.32
Autochthonous	9	18	6 ± 1.1 ^ab^	6.03 ± 0.74 ^ab^	610.8 ± 160.3	790.9 ± 81.24	3.25 ± 0.31 ^ab^	3.18 ± 0.20 ^ab^	2.87 ± 0.35	3.12 ± 0.19	65.56 ± 7.38	72.41 ± 3.11

Different letters indicate statistically significant differences between breeds (*p* < 0.05, One Way ANOVA).

## Data Availability

Not applicable.

## References

[1] Coulter G.H., Foote R.H. (1979). Bovine testicular measurements as indicators of reproductive performance and their relationship to productive traits in cattle: A review. Theriogenology.

[2] Schillo K.K., Hall J.B., Hileman S.M. (1992). Effects of nutrition and season on the onset of puberty in the beef heifer. J. Anim. Sci..

[3] Tanaka M., Hennebold J.D., Miyakoshi K., Teranishi T., Ueno K., Adashi E.Y. (2003). The generation and characterization of an ovary-selective cDNA library. Mol. Cell Endocrinol..

[4] Adamczewski J.Z., Fargey P.J., Laarveld B., Gunn A., Flood P.F. (1998). The influence of fatness on the likelihood of early-winter pregnancy in muskoxen (*Ovibos moschatus*). Theriogenology.

[5] Alexander J.H. (2008). Bull breeding soundness evaluation: A practitioner’s perspective. Theriogenology.

[6] Coulter G.H., Kozub G.C. (1989). Efficacy of methods used to test fertility of beef bulls used for multiple-sire breeding under range conditions. J. Anim. Sci..

[7] Barth A.D., Arteaga A.A., Brito L.F., Palmer C.W. (2004). Use of internal artificial vaginas for breeding soundness evaluation in range bulls: An alternative for electroejaculation allowing observation of sex drive and mating ability. Anim. Reprod. Sci..

[8] Ministerio de Agricultura, Pesca y Alimentación. Registro: Sistema Integral de Trazabilidad Animal (SITRAN). https://www.mapa.gob.es/es/ganaderia/temas/trazabilidad-animal/registro/default.aspx.

[9] Parkinson T.J. (2004). Evaluation of fertility and infertility in natural service bulls. Vet. J..

[10] Sanderson M.W., Gnad D.P. (2002). Biosecurity for reproductive diseases. Vet. Clin. N. Am. Food Anim. Pract..

[11] Frey C.F., Regidor-Cerrillo J., Marreros N., García-Lunar P., Gutiérrez-Expósito D., Schares G., Dubey J.P., Gentile A., Jacquiet P., Shkap V. (2016). Besnoitia besnoiti lytic cycle in vitro and differences in invasion and intracellular proliferation among isolates. Parasit Vectors.

[12] Martinez-Ibeas A.M., Power C., McClure J., Sayers R.G. (2015). Prevalence of BoHV-1 seropositive and BVD virus positive bulls on Irish dairy farms and associations between bull purchase and herd status. Ir. Vet. J..

[13] Waldner C.L., Kennedy R.I. (2008). Associations between health and productivity in cow-calf beef herds and persistent infection with bovine viral diarrhea virus, antibodies against bovine viral diarrhea virus, or antibodies against infectious bovine rhinotracheitis virus in calves. Am. J. Vet. Res..

[14] Barlow R.M., Nettleton P.F., Gardiner A.C., Greig A., Campbell J.R., Bonn J.M. (1986). Persistent bovine virus diarrhoea virus infection in a bull. Vet. Rec..

[15] Fray M.D., Paton D.J., Alenius S. (2000). The effects of bovine viral diarrhoea virus on cattle reproduction in relation to disease control. Anim. Reprod. Sci..

[16] Viet A.F., Fourichon C., Seegers H. (2007). Review and critical discussion of assumptions and modelling options to study the spread of the bovine viral diarrhoea virus (BVDV) within a cattle herd. Epidemiol. Infect..

[17] Meyling A., Jensen A.M. (1988). Transmission of bovine virus diarrhoea virus (BVDV) by artificial insemination (AI) with semen from a persistently-infected bull. Vet. Microbiol..

[18] Givens M.D., Riddell K.P., Edmondson M.A., Walz P.H., Gard J.A., Zhang Y., Galik P.K., Brodersen B.W., Carson R.L., Stringfellow D.A. (2009). Epidemiology of prolonged testicular infections with bovine viral diarrhea virus. Vet. Microbiol..

[19] Bielanski A., Dubuc C. (1994). In vitro fertilization and culture of ova from heifers infected with bovine herpesvirus-1 (BHV-1). Theriogenology.

[20] Kirkland P.D., Richards S.G., Rothwell J.T., Stanley D.F. (1991). Replication of bovine viral diarrhoea virus in the bovine reproductive tract and excretion of virus in semen during acute and chronic infections. Vet. Rec..

[21] Sayers R.G. (2017). Associations between exposure to bovine herpesvirus 1 (BoHV-1) and milk production, reproductive performance, and mortality in Irish dairy herds. J. Dairy Sci..

[22] Statham J.M., Randall L.V., Archer S.C. (2015). Reduction in daily milk yield associated with subclinical bovine herpesvirus 1 infection. Vet. Rec..

[23] Acuña C.M. (2008). Manejo de Los Toros; Sitio Argentino de Produccion Animal. http://www.produccion-animal.com.ar/informacion_tecnica/cria_toros/36-manejo.pdf.

[24] De Cría G.T.E.R. (2014). El uso de toros de muy alta capacidad de servicio para obtener una alta tasa de gestación temprana en rodeos de cría. Sitio argentino de producción animal. http://www.produccion-animal.com.ar/informacion_tecnica/cria_toros/75-toros_de_muy_alta_capacidad.pdf.

[25] Barth A. (1995). Evaluation of frozen bovine semen by the veterinary practitioner. Proceedings of the Bovine Short Course.

[26] Kirkland P.D., Mackintosh S.G., Moyle A. (1994). The outcome of widespread use of semen from a bull persistently infected with pestivirus. Vet. Rec..

[27] Voges H., Horner G.W., Rowe S., Wellenberg G.J. (1998). Persistent bovine pestivirus infection localized in the testes of an immuno-competent, non-viraemic bull. Vet. Microbiol..

[28] McClurkin A.W., Coria M.F., Cutlip R.C. (1979). Reproductive performance of apparently healthy cattle persistently infected with bovine viral diarrhea virus. J. Am. Vet. Med. Assoc..

[29] Revell S.G., Chasey D., Drew T.W., Edwards S. (1988). Some observations on the semen of bulls persistently infected with bovine virus diarrhoea virus. Vet. Rec..

[30] El-Mohamady R.S., Behour T.S., Rawash Z.M. (2020). Concurrent detection of bovine viral diarrhoea virus and bovine herpesvirus-1 in bulls’ semen and their effect on semen quality. Int. J. Vet. Sci. Med..

[31] Brock K., Grooms D., Daniel Givens M., Ridpath S.M.G.J.F. (2005). Reproductive Disease and Persistent Infections. Bovine Viral Diarrhea Virus: Diagnosis, Management, and Control.

[32] Rahman M.M., Kabir A., Khaton R., Rahman S., Ahmed S., Sardar M.J.U., Islam M.H., Rahman M.S., Islam M.R. (2017). The dynamics of Bovine Viral Diarrhea Virus (BVDV) infection and possible impacts on Cattle reproduction. Bangladesh Livest. J..

[33] Cowley D.J., Clegg T.A., Doherty M.L., More S.J. (2011). Aspects of bovine herpesvirus-1 infection in dairy and beef herds in the Republic of Ireland. Acta Vet. Scand..

[34] Raaperi K., Orro T., Viltrop A. (2014). Epidemiology and control of bovine herpesvirus 1 infection in Europe. Vet. J..

[35] Makoschey B., Zehle H.H., Bussacchini M., Valla G., Pálfi V., Földi J. (2007). Efficacy of a live bovine herpesvirus type 1 marker vaccine under field conditions in three countries. Vet. Rec..

[36] Timlin C., Hungerford L., Redifer T., Currin J.F., Rodrigues Gomes Mercadante V. (2019). A restrospective analysis of bull:cow ratio effects on pregnancy rates of beef cows previously enrolled in fixed-time artificial insemination protocols. J. Anim. Sci..

